# China's standardized residency training in the context of public health: a controversial decade—Signs of hope?

**DOI:** 10.3389/fpubh.2025.1552075

**Published:** 2025-05-07

**Authors:** Yuan Liu, Linghe Qiu, Yugang Xue, Jianhong Wu, Na Li

**Affiliations:** ^1^Outpatient Department, Affiliated Mental Health Center of Jiangnan University, Wuxi Central Rehabilitation Hospital, Wuxi, Jiangsu, China; ^2^Pharmacy Department, The Affiliated Wuxi People's Hospital of Nanjing Medical University, Wuxi Medical Center, Nanjing Medical University, Wuxi, Jiangsu, China; ^3^Pharmacy Department, Taizhou People's Hospital, Taizhou, Jiangsu, China

**Keywords:** Standardized Residency Training (SRT), dual-track, Equal Treatment for Two Categories, China, public health

A decade has passed since the full implementation of the Standardized Residency Training (SRT) system in China (1). In 2014, China's health authorities officially released the “Guiding Opinions on Establishing the Standardized Residency Training System” and the first batch of SRT bases, initiating a nationwide rollout of the SRT program in the following year (2). Since then, SRT has taken on the role of providing medical graduates with the necessary knowledge and skills before fully entering the profession, playing a key role in promoting the standardization and homogenization of national public healthcare (3). It can be said that the SRT system shapes the future of China's healthcare.

However, this system has faced numerous doubts and challenges over the past decade. Heavy workloads, minimal learning gains, and low salaries have become common issues for residents in China (4). Additionally, the dual-track training system has significantly impacted the residency framework (5). Although both tracks involve a three-year training period, whether residents obtain the “four certificates” [Master of Medicine (MM) degree, graduation certificate, SRT certificate, and medical license] after SRT is the key distinction between the MM program, postgraduate residency training for Master of Science (MSc) in Medicine and Bachelor's degree graduates (6). MM students must complete their degree during the SRT period and can obtain all four certificates upon graduation. In contrast, those with a Bachelor's degree can only receive the SRT certificate and medical license. For MSc graduates, despite having completed 3 years of research training, an additional 3 years of SRT is required (7). Experiencing similar training but receiving different outcomes, this dual-track system increases the complexity of managing residents and complicates compensation and career development. Moreover, there is a significant disparity in compensation between the two tracks. Postgraduate residency training is considered employment and generally offers higher compensation and benefits compared to SRT for MM students, who are still classified as students. Although policy incentives have significantly shortened the time required for MM students to obtain the four certificates, this has also increased their pressure (8).

This issue is expected to be thoroughly alleviated. Recently, the National Health Commission (NHC) and the Ministry of Education jointly issued a document emphasizing the previously proposed policy of “Equal Treatment for Two Categories” (9), which has sparked vigorous discussions among medical professionals and students. The NHC stated that clinical physicians with a Bachelor's degree who have completed SRT will receive equal treatment in terms of recruitment, title promotion, job placement, and compensation compared to MM students. Additionally, former recent graduates will be treated the same as current-year graduates after completing their SRT.

This policy is expected to have a profound impact on the cultivation of medical talent in China. First, it will accelerate the integration of the dual-track SRT system, providing Bachelor's degree residents unprecedented advantages (10). They will benefit from higher compensation, face less pressure, and will not need to take National Entrance Examination for Postgraduates (NEEP). Additionally, according to the policy, they can be awarded an MM degree if they meet national requirements. As more medical students and hospitals reach a consensus on postgraduate SRT for Bachelor's degree holders, fewer students will opt for MM programs. Over time, this will likely reduce the number of such postgraduate students and indirectly ease the SRT pressure on MM students. Even if Bachelor's degree residents do not meet the requirements for an MM degree during SRT, they can still apply for jobs with an equivalent academic qualification, making the MM program an optional path. A similar reduction in enrollment is expected for the MSc program. Undergraduates who did not achieve the necessary scores for an MM program and instead enrolled in an MSc program may choose to enter SRT directly, as it offers a shorter pathway and the possibility of obtaining an MM degree. With the comprehensive implementation of the Sanming Medical Reform (doctor annual salary system) (11), the break from paper-centric academic promotions, and the liberalization of foreign medical institutions in China, the appeal of a purely research-focused MSc degree will significantly diminish. Second, in China's current economic context, having the status of a “recent graduate” is a significant advantage in the job market, particularly for hospital recruitment. The “Equal Treatment for Two Categories” policy provides Bachelor's degree holders in SRT with equal status to MM graduates.

It is foreseeable that this policy may effectively alleviate burnout in SRT for a certain period. Currently, a significant proportion of Chinese physician experience burnout related to SRT, with contributing factors including work pressure, compensation issues, as well as systemic and policy-related factors (12). Moreover, some Bachelor's degree graduates face failure during NEEP, leading to feelings of disappointment and confusion during their SRT, which may negatively impact their sense of self-efficacy and career planning (13). Fortunately, the new policy is expected to enhance the overall benefits for physicians with a Bachelor's degree, reducing their financial stress while improving professional competitiveness and job satisfaction (14). By “leveling” the educational discrimination in employment, this policy encourage undergraduates to directly participate in SRT, enabling them to enter clinical frontline work earlier. This approach addresses the shortage of medical talent and bridges the gap in China's disorganized clinical medicine programs (15), where SRT graduates are treated on par with those holding an MM degree. By increasing the number of residents and granting equal treatment, it helps alleviate occupational burnout among SRT participants. Furthermore, the “Equal Treatment for Two Categories” policy may accelerate salary reforms within the industry, driving comprehensive adjustments to title and salary structure with a stronger focus on practical skills and job requirements.

In addition, by establishing this policy, the government can accelerate the training and employment of medical students, reducing the over-reliance on academic research-focused training models and promoting medical education that is more aligned with actual healthcare needs (16). Rather than investing excessive resources into research-oriented training models, medical students should focus more on practice-oriented educational approaches (2) to more quickly become healthcare professionals that meet specific needs. For eligible medical students, an MM degree can still be awarded. This policy marks a shift in SRT—traditionally the equalizer for residents across different regions (17)—toward a greater emphasis on clinical training for physicians, prioritizing the development of clinical skills over purely academic education. This strategy, which downplays degree-based education while strengthening vocational training, sends a strong signal that healthcare is the priority. This shift is expected to enhance the country's ability to provide services in public health areas such as disease prevention and health management, ensure the sustainability of the healthcare system, and improve overall public health service levels.

Although this policy provides more opportunities for medical graduates with a Bachelor's degree, it may also bring some negative effects. First, an over-reliance on the SRT pathway by undergraduates may limit the breadth of their academic development. Medical students without research experience may lack competitiveness in the field of scientific research (18), affecting their future career development. Second, as more undergraduates choose SRT, this may lead to an over-concentration of medical education, reducing the diversity of different educational paths (such as MM and MSc degrees), which in turn could affect the comprehensiveness and innovation of the medical profession (19). Additionally, this policy may dilute the value of the MM degree, as Bachelor's degree holders who complete SRT are treated equally to those with an MM degree, leading to increased competition between the two pathways. The distinction between the two may gradually blur, affecting the differentiation between degree education and SRT (20).

It is important to note that China's rapid transition from a planned economy to a market-driven economy has led to imbalanced development between urban and rural areas, particularly in healthcare (21). Although the Chinese government has implemented a series of measures, including the New Rural Cooperative Medical Scheme (NRCMS), to mitigate this gap, concerns still persist (22). One of the key challenges in China's healthcare system is the imbalance and insufficiency of healthcare development across regions. However, the “Equal Treatment for Two Categories” policy may exacerbate this phenomenon, particularly in remote areas with scarce public health resources. According to the policy, Bachelor's degree graduates gain the same competitiveness as those with an MM degree after completing SRT. This may exacerbate talent loss in underdeveloped regions. Importantly, a core task of public health work is to improve grassroots medical care (23). Therefore, how to balance the allocation of medical resources and the mobility of personnel in underdeveloped areas is a key direction that the government needs to guide. Perhaps requiring beneficiaries of the new policy to provide targeted services in underdeveloped regions for a certain period could be an effective solution (24). Finally, China's uneven regional development may lead to inconsistent implementation of the new policy across different areas, particularly in underdeveloped regions where it may be difficult to access equal treatment or high-quality SRT opportunities.

With the gradual reform of China's healthcare system, the advancement of the “Equal Treatment for Two Categories” policy and its alignment with public health policies will have a profound impact on the nation's overall health strategy. We sincerely hope that this policy will be strongly implemented to better support medical students' SRT pathways and career development, and to optimize the SRT system in China. Subsequent evaluation measures should be promptly followed up to assess the outcomes of the policy.
